# Anticolon Cancer Properties of Pyrazole Derivatives Acting through Xanthine Oxidase Inhibition

**DOI:** 10.1155/2021/5691982

**Published:** 2021-07-05

**Authors:** Abdulrhman Alsayari, Yahya I. Asiri, Abdullatif Bin Muhsinah, Mohd. Zaheen Hassan

**Affiliations:** College of Pharmacy, King Khalid University, Abha 61441, Saudi Arabia

## Abstract

**Background:**

Pyrazoles are an interesting class of compounds showing potent anticancer activities. Our previous studies have demonstrated the potent anticancer activity of pyrazole analogues. Therefore, we focused on developing anticancer agents through structure optimization of the pyrazolyl lead molecule.

**Methods:**

The pyrazole derivatives were prepared by the appropriate synthetic protocols. The antiproliferative activities were evaluated using a sulforhodamine B assay against three cancer cell lines. In vitro and in silico molecular docking studies employing xanthine oxidase were used to explore the mechanism by which pyrazole derivatives exert anticancer effects.

**Results:**

One of the pyrazole derivatives demonstrated the greatest promise as an anticancer agent against the human colon cancer cell line (IC_50_ 4.2 *μ*M), with a potent xanthine oxidase inhibitory activity (IC_50_ 0.83 *μ*M).

**Conclusion:**

In summary, our findings suggest that these pyrazolyl analogues containing a pyridine nucleus could serve as a promising lead molecule in the development of novel anticancer agents.

## 1. Introduction

Cancer is one of the four major types of noncommunicable diseases, along with cardiovascular disease, diabetes, and chronic respiratory diseases [1]. It is a disease in which abnormal cells divide without control and can invade nearby tissues [2]. The cancer burden continues to grow globally, with 18.1 million new cases and 9.6 million deaths in the preceding year [3]. Among males, the most prevalent cancers are lung, prostate, colorectal, stomach, and liver cancers, whereas among females, breast, colorectal, lung, cervical, and thyroid cancers are the most common [4]. This growing universal cancer burden is exerting huge physical, emotional, and financial strains on society. Moreover, chemotherapy resistance and its associated serious side effects, even with the targeted therapies, continue to be a major concern for oncologists [5]. Therefore, it is imperative that we develop safer and more effective anticancer agents acting through novel mechanisms and target only the cancer cells.

Pyrazoles are a promising scaffold for many anticancer agents. A number of clinical anticancer therapeutics, such as crizotinib, ruxolitinib, niraparib, encorafenib, and darolutamide, currently consist of a pyrazole moiety [6]. Therefore, in the past decades, a large number of pyrazolyl analogues were synthesized and tested as anticancer agents [7]. Our research group focused on the design and synthesis of pyrazole derivatives functionalized with aryldiazo pyrazoles. In our previous research, aryldiazo pyrazoles showed a very promising anticancer activity against MCF-7, HepG2, and HCT-116 cell lines. For instance, 4-[2-(4-nitrophenyl)hydrazono]-2-pyrazolin-5-one, as a lead molecule, showed an inhibition of cell proliferation with an IC_50_ value in the range of 0.2–3.4 *μ*M [8]. Encouraged by these results, we further optimized the lead molecule through different substitutions on the aryl ring. These compounds were synthesized using our previously developed rapid one-pot three-component condensation method for the synthesis of pyrazolyl analogues [8] and then evaluated the resulting molecules for their anticancer potential.

Since cancer and oxidative stress share some aspects of their underlying pathophysiology, some antioxidants are equally effective as anticancer agents [9]. The cellular redox process produces reactive oxygen species (ROS) as by-products. These ROS exert beneficial effects on cellular response at low levels, whereas they can be deleterious and may cause DNA damage and cancer at high levels. Antioxidants have been shown to act as “free radical scavenger” by preventing damages caused by ROS and thus exert a protective effect against several different types of cancers [10]. Moreover, xanthine oxidase (XO) overexpression has been linked to the progress of oncogenesis through the generation of cytotoxic reactive oxygen species (ROS). Therefore, XO has been identified as a potential target for anticancer agents [11]. Recently, many studies have also reported that pyrazole derivatives are very promising XO inhibitors [12]. Therefore, these promising cytotoxic compounds were also subjected to further investigation for their XO inhibitory activities.

## 2. Materials and Methods

Pyrazolyl analogues **1** and **2** were synthesized using our previously described procedures [8]. These compounds were evaluated for their in vitro anticancer activity against HepG2, HCT-116, and MCF-7 cell lines by the sulforhodamine B assay (SRB).

### 2.1. Cell Culture

Human hepatocellular carcinoma cell line (HepG-2, ATCC HB-8065), colorectal adenocarcinoma cell line (HCT-116, ATCC CCL-247), and breast adenocarcinoma cell line (MCF-7, ATCC HTB-22) were obtained from the American type culture collection (ATCC). Cells were maintained in RPMI-1640 supplemented with (100 *μ*g/ml) penicillin (100 units/ml) and heat-inactivated fetal bovine serum (10% v/v) in a humidified, 5% (v/v) CO_2_ atmosphere at 37°C [13].

### 2.2. Cytotoxicity Assessment

The cytotoxicity of the different compounds was tested against human tumor cells using the sulforhodamine B assay (SRB). Healthy growing cells were cultured in a 96-well tissue culture plate (3000 cells/well) for 24 hours to allow attachment of the cells to the plate before treatment with the tested compounds. Cells were exposed to five different concentrations of each compound (0.01, 0.1, 1, 10, and 100 *μ*M/ml); untreated cells (control) were also added. Triplicate wells were incubated with different concentrations for 72 h and subsequently fixed with TCA (10% w/v) for 1 h at 4°C. After several washings, cells were stained with 0.4% (w/v) SRB solution for 10 min in a dark place. Excess stain was washed with 1% (v/v) glacial acetic acid. After drying overnight, the SRB-stained cells were dissolved with tris-HCl and the color intensity was measured in a microplate reader at 540 nm. The linear relationship between the viability percentage of each tumor cell line and the compounds' concentrations was analyzed to obtain IC_50_ (dose of the drug which reduces survival to 50%) using SigmaPlot 12.0 software [14].

### 2.3. Xanthine Oxidase Assay

These compounds were further evaluated for their XO inhibitory potential by measuring the formation of uric acid concentration spectrophotometrically at 292 nm using a method previously described [15]. Solutions of the enzyme-drug complex were prepared by mixing freshly prepared XO enzyme solution (0.5 mL) with 1.0 mL solution of test compounds (at concentrations of 100, 50, 25, 12.5, 6.25, and 3.12 *μ*M, dissolved in DMSO and diluted with a buffer) in a phosphate buffer (1.5 mL of 50 mM; pH 7.4) and incubated at 25°C for 15 min. Subsequently, 0.45 mL of the xanthine solution substrate was added and incubated at 25°C for another 30 min. The enzymatic reaction was stopped by adding 1 mL HCl (1 M). Allopurinol was used as a standard, and a phosphate buffer was substituted for xanthine as a blank. The XO inhibition was expressed as % inhibition and compared to the control with the following formula: % inhibition = [(Ac − As)/Ac] × 100, where Ac indicates the absorbance of the control sample and As is the absorbance of the treated sample. Readings were taken in triplicate and represented as IC_50_ ± SD. IC_50_ values were calculated using GraphPad Prism 8 software.

### 2.4. Molecular Docking Studies

Molecular docking studies were performed using AutoDock v. 4.2.2 to identify appropriate binding modes and conformation of the ligand molecules. The crystal structure of xanthine dehydrogenase (PDB code: 3BDJ, resolution: 2.0 Å) was retrieved from the RCSB Protein Data Bank as a PDB format [16]. The structures of all the ligands were drawn using ChemDraw Ultra 13.0 and converted into 3D structures using HyperChem Pro 8.0 software (http://www.hyper.com). AutoDock tools (ADT) version 1.5.6 (http://www.autodock.scrips.edu) was used to prepare the molecular docking. The active site was considered as a rigid molecule, while the ligands were treated as being flexible. Using default parameters, grid-based docking studies were carried out and docking was performed on all compounds using the standard ligand oxipurinol. The best binding conformation was selected from the docking log (.dlg) file for each ligand, and further interaction analysis was performed using PyMOL and Discovery Studio Visualizer 4.0.

## 3. Results and Discussion

**Figure 1:**
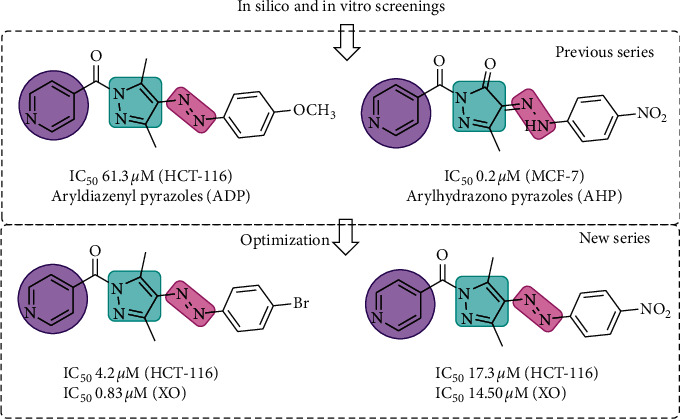
Design strategy and lead optimization of newer pyrazole analogues, showing the different bioactive pharmacophores essential for the anticancer activity.

Based on the encouraging results of 1-isonicotinoyl-3-methyl-4-[2-(4-nitrophenyl)hydrazono]-2-pyrazolin-5-one (IC_50_ 0.2–3.4 *μ*M) as a potential anticancer lead molecule, we focused our attention on optimizing the anticancer potential of arylhydrazono-pyrazole derivatives with different substitutions at the phenyl ring (Figure 1). The newly synthesized compounds **1** and **2** were evaluated for the antiproliferative activity against three human tumor cell lines, namely, breast cancer (MCF-7), hepatocellular carcinoma (HepG2), and colorectal carcinoma (HCT-116), by the sulforhodamine B (SRB) assay. The SRB assay is the “gold-standard” assay and is extensively used in the high-throughput screening program at the National Cancer Institute (NCI), USA. The SRB uses an aminoxanthene dye, which readily stains the cells through binding with the basic amino acids of cellular proteins. The SRB assay estimates the dye released from the stained cells after washing as stoichiometric proportions of cellular mass. In Table 1, the results of the SRB assay of the titled compounds are presented as 50% growth inhibitory concentration (IC_50_) values. The IC_50_ values were determined by interpolation from dose-response curves. From the results, it was observed that most of the newly synthesized pyrazoles showed excellent to moderate antiproliferative activity against the three cell lines, especially against colorectal carcinoma (HCT-116). Compound **1** effectively inhibited cell growth at IC_50_ values of 17.8 ± 0.5, 4.4 ± 0.4, and 4.2 ± 0.2 *μ*M against the breast cancer (MCF-7), hepatocellular carcinoma (HepG2), and colorectal carcinoma (HCT-116) cell lines, respectively. The anticancer activity of compound **1** was comparable to the standard drug doxorubicin against the HepG2 (3.9 ± 0.06) and HCT-116 (4.4 ± 0.04) cell lines, but 3.7 times less potent in the MCF-7 (4.7 ± 0.08) cell lines. Compound **2** was found to be moderately effective, with IC_50_ values of 94.2 ± 0.3, 34.6 ± 2.6, and 17.3 ± 0.5 *μ*M against the MCF-7, HepG2, and HCT-116 cell lines, respectively. Our previous studies have shown that unsubstituted phenyl derivative of aryldiazenyl pyrazole had a poor antiproliferative activity (IC_50_ > 100 *μ*M) against all three cell lines [8]. This finding indicates that substitution with electron withdrawing groups such as bromo and nitro groups significantly improved the anticancer potential of aryldiazenyl pyrazole derivatives.

Pyrazolyl analogues have been reported as promising anticancer agents acting through xanthine oxidase (XO) inhibition [12]. The promising anticancer properties of pyrazole derivatives **1** and **2** further prompted us to assess their XO inhibitory activity to gain insight into the mechanism of the anticancer activity of these pyrazole derivatives. The assessment of the XO inhibitory activity was carried out spectrophotometrically by measuring the formation of uric acid at 290 nm. The results indicated that compound **1** (IC_50_ 0.83 ± 1.36 *μ*M) exhibited significant XO inhibition, 18.03 times more potent than the standard drug allopurinol (IC_50_ 14.97 ± 1.61 *μ*M) (Table 2), whereas compound **2** was equipotent (IC_50_ 14.50 ± 2.25 *μ*M) to allopurinol. These results confirm the XO inhibitory action of these compounds and suggest the possible influence of XO inhibition in the anticancer activity of pyrazoles. However, further kinetic studies are required to establish a more detailed mode of enzyme inhibition of these compounds.

**Figure 2:**
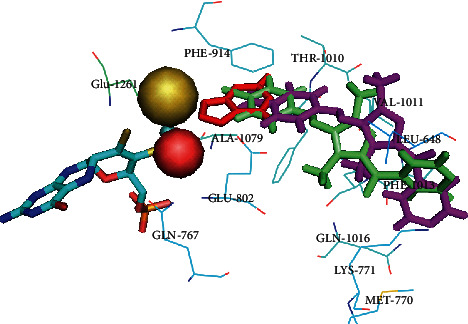
3D view of docked pose of pyrazolyl analogues **1** (lime) and **2** (magenta), with oxipurinol (red) at the vicinity of the molybdopterin cofactor.

Encouraged by the results of the in vitro anticancer and XO inhibitory activities of the newly synthesized pyrazolyl derivatives, molecular docking studies were carried out on xanthine dehydrogenase (XDH) using AutoDock. Both the enzymes XO and XDH are interconvertible forms of the same enzyme, known as xanthine oxidoreductase (XOR), with only slight differences in the FAD binding domain. The crystal structure of bovine milk XDH bound with oxipurinol was used in the current study and retrieved from the raw PDB structure 3BDJ, which has 90% similarity to the human liver enzyme [16]. Oxipurinol is a potent XOR inhibitor acting through a tight binding to the reduced molybdenum ion (Mo^4+^) of the molybdopterin cofactor, which is essential for the mechanism-based inhibition. Furthermore, it forms hydrogen bonds with Glu802, Arg880, and Glu1261 in the active site of the enzyme (Figure 2). These binding interactions are concordant with previous reports. The results of the molecular docking studies of the two pyrazole derivatives revealed that they occupy the same narrow channel that leads towards the molybdenum center in the active site; however, they do not interact with the cofactor. The binding free energy of these two compounds was in the range of −6.1 to −7.6 kcal/mol, indicating sufficient affinity between the enzyme and inhibitors (Table 2). The key interacting residues at the active site include Leu648, Phe649, Gln767, Met770, Glu802, Arg880, Phe914, Phe1009, Thr1010, Val1011, Phe1013, Leu1014, Glu1016, and Glu1261 (Figure 2). The results of the docking studies are consistent with the in vitro study and indicate the strong inhibitory activities of pyrazole derivatives against XO. Moreover, these data also indicate that the anticancer activity of pyrazolyl analogues might be due to XO inhibition.

After obtaining interesting results with arylhydrazono-pyrazole analogues, different substitutions were made at the phenyl ring for optimizing the template. The analogues **1** and **2** were prepared by using the method described earlier [8]. Their antiproliferative activities were evaluated in comparison with the unsubstituted phenyl derivative (IC_50_ > 100 *μ*M), and compound **1** was about >22 folds more active than its unsubstituted derivative in the HepG2 and HCT-116 cell lines. These findings suggested that the electron withdrawing groups at the phenyl ring might be important for high potency. Furthermore, HCT-116 was the most sensitive cell line against these pyrazolyl analogues.

In conclusion, compound **1** emerged as a promising anticancer agent effective against the colorectal carcinoma cell lines (IC_50_ 4.2 *μ*M) acting through xanthine oxidase inhibition (IC_50_ 0.83 *μ*M). Results of in vitro and in silico xanthine oxidase inhibitory activities revealed that the anticancer activity of these pyrazolyl analogues might be due to XO inhibition.

## Figures and Tables

**Table 1:** The IC_50_ (*μ*M) of pyrazolyl derivatives against different tumor cell lines.

Compound	*R*	IC_50_ (*μ*M)		
		MCF-7	HepG2	HCT-116
1	Br	17.8 ± 0.5	4.4 ± 0.4	4.2 ± 0.2
2	NO_2_	94.2 ± 0.3	34.6 ± 2.6	17.3 ± 0.5
Doxorubicin	—	4.7 ± 0.08	3.9 ± 0.06	4.4 ± 0.04

**Table 2:** Xanthine oxidase inhibition activities of pyrazole derivatives (1 and 2).

Compound	Binding free energy (kcal/mol)	IC_50_ (*μ*M)
1	−7.6	0.83 ± 1.36
2	−6.1	14.50 ± 2.25
Allopurinol	—	14.97 ± 1.61

## Data Availability

The data used to support the findings of this study are available from the corresponding author upon request.
